# Playing the computer game Tetris prior to viewing traumatic film material and subsequent intrusive memories: Examining proactive interference

**DOI:** 10.1016/j.jbtep.2015.11.004

**Published:** 2016-12

**Authors:** Ella L. James, Alex Lau-Zhu, Hannah Tickle, Antje Horsch, Emily A. Holmes

**Affiliations:** a University of Oxford, Department of Psychiatry, Warneford Hospital, Oxford, OX3 7JX, UK; b University of Oxford, Department of Experimental Psychology, South Parks Road, Oxford, OX1 3UD, UK; c University of Lausanne, Department of Child and Adolescent Psychiatry, Lausanne, Switzerland; d University of Lausanne, Department of Neonatology, Lausanne, Switzerland; e University of Lausanne, Department of Obstetrics and Gynecology, Lausanne, Switzerland; f Medical Research Council [MRC] Cognition and Brain Sciences Unit, 15 Chaucer Road, Cambridge CB22 7EF, UK

**Keywords:** Intrusive memory, Trauma film paradigm, Working memory task interference, Tetris, Post-traumatic stress disorder, Memory consolidation, Trauma prevention

## Abstract

**Background and objectives:**

Visuospatial working memory (WM) tasks performed concurrently or after an experimental trauma (traumatic film viewing) have been shown to reduce subsequent intrusive memories (concurrent or retroactive interference, respectively). This effect is thought to arise because, during the time window of memory consolidation, the film memory is labile and vulnerable to interference by the WM task. However, it is not known whether tasks before an experimental trauma (i.e. proactive interference) would also be effective. Therefore, we tested if a visuospatial WM task given before a traumatic film reduced intrusions. Findings are relevant to the development of preventative strategies to reduce intrusive memories of trauma for groups who are routinely exposed to trauma (e.g. emergency services personnel) and for whom tasks prior to trauma exposure might be beneficial.

**Methods:**

Participants were randomly assigned to 1 of 2 conditions. In the *Tetris* condition (n = 28), participants engaged in the computer game for 11 min immediately before viewing a 12-min traumatic film, whereas those in the Control condition (n = 28) had no task during this period. Intrusive memory frequency was assessed using an intrusion diary over 1-week and an Intrusion Provocation Task at 1-week follow-up. Recognition memory for the film was also assessed at 1-week.

**Results:**

Compared to the Control condition, participants in the *Tetris* condition did not report statistically significant difference in intrusive memories of the trauma film on either measure. There was also no statistically significant difference in recognition memory scores between conditions.

**Limitations:**

The study used an experimental trauma paradigm and findings may not be generalizable to a clinical population.

**Conclusions:**

Compared to control, playing *Tetris before* viewing a trauma film did not lead to a statistically significant reduction in the frequency of later intrusive memories of the film. It is unlikely that proactive interference, at least with this task, effectively influences intrusive memory development. WM tasks administered during or after trauma stimuli, rather than proactively, may be a better focus for intrusive memory amelioration.

## Highlights

• When trauma is predictable (e.g. for emergency workers) preventative interventions delivered *before* trauma would be useful.
• Could a visuospatial working memory (WM) task before analogue trauma reduce intrusions via proactive interference?
• Participants played *Tetris* or had no task prior to viewing a traumatic film.
• There was no statistically significant difference in intrusive memory frequency of the trauma film between both groups.
• WM tasks delivered during/after trauma stimuli, rather than before, may be a better focus for intrusive memory amelioration.

## 1 Introduction

The presence of recurrent, involuntary and intrusive distressing memories of a traumatic event are common in the early aftermath of psychological trauma and are a hallmark of both Acute Stress Disorder (ASD) and post-traumatic stress disorder (PTSD; DSM-5, American Psychiatric Association, 2013). Such negative, intrusive memories are proposed to occur due to excessive perceptual (sensory) processing during a trauma (Brewin, 2014, Ehlers and Clark, 2000) resulting in sensory-based (predominately visual) images of the trauma that intrude into mind spontaneously.

In this special issue, we pay tribute to Van den Hout's pioneering work. Among so many other things, his work with colleagues provides an experimental psychopathology test of the above clinical theory. An experiment using the trauma film paradigm revealed that the style in which an individual processes a traumatic event influences later intrusive memories (Kindt, Van den Hout, Arntz, & Drost, 2008). Participants who were instructed to engage in conceptually-driven processing, relative to those who engaged in sensory-based, data-driven processing, reported fewer intrusive memories to the traumatic film (Experiment 1) and had a reduced tendency to suppress intrusive memories (Experiment 2). Results may also indicate that tasks which interfere with data-driven processing (e.g. sensory-perceptual, visuospatial-based tasks) may be beneficial in reducing the occurrence of intrusive memories (for a diagramatic summary see, Holmes & Bourne, 2008).

Understanding possible processes to prevent later trauma symptoms is an important task for experimental psychopathology research. Many individuals are routinely exposed to events that are stressful and traumatic, for example in the work place (e.g. emergency services, military personnel) or in hostile environments (e.g. war zones) and are at risk of developing ASD and PTSD (Breslau et al., 1998, Bryant et al., 2012, Kessler et al., 1995). In such instances where exposure to traumatic events is predictable, the development of pre-emptive strategies (e.g. methods that can be used before entering a traumatic situation) to reduce traumatic stress symptoms, such as intrusive memories, may be clinically useful. A recent line of our work has tested the delivery of cognitive tasks during (Bourne et al., 2010, Holmes et al., 2004) and after traumatic stimuli (Deeprose et al., 2012, Holmes et al., 2009, Holmes et al., 2010, James et al., 2015), but not before. Thus, it remains to be tested whether such cognitive tasks can interfere when delivered prior to the encoding of traumatic stimuli.

Memory impairment as a consequence of interference has been widely researched in the (non-clinical) memory literature, where interference is proposed to be a primary source of forgetting (e.g. Dewar et al., 2007, Muller and Pilzecker, 1900). Retroactive and proactive interference are two forms of interference (Wixted, 2004). Retroactive interference describes incidences where *new* information impedes the recall of previously learnt events (i.e. interference after the event). In contrast, proactive interference describes instances where *prior* information interferes with the recall of more recent events (i.e. interference before the event). Third, concurrent interference effects can occur when two tasks are undertaken simultaneously leading to impairment compared to if tasks were undertaken separately, as has been investigated using dual task paradigms (Silver et al., 2013). Critical to the current study hypothesis, memory interference theory suggests that tasks, regardless of their order presentation, can interfere with one another (Wixted, 2004). A memory interference perspective thus holds relevance to experimental psychopathology research on emotional memories, for example in considering the order and impact of cognitive tasks which may (or may not) interfere with such memories. We next discuss studies involving concurrent interference from Van den Hout and colleagues, then retroactive interference with cognitive tasks administered after trauma film viewing, and then note a gap in knowledge regarding proactive interference and trauma memory, with a fuller discussion of proactive interference from the non-clinical literature.

Marcel Van den Hout has pioneered research which suggests that certain working memory (WM) tasks undertaken concurrently with recalling memories of distressing events can reduce the vividness and/or emotionality of that memory. For example, Van den Hout, Muris, Salemink, and Kindt (2001) asked participants to recall a negative autobiographical memory whilst either engaging in a concurrent eye-movement task (tracking the experimenter's rapid hand movements), a concurrent rhythmic tapping task or no concurrent task. Results showed reduced emotionality and vividness for the memory in the eye-movement condition only. A further elegant clinical experiment with patients with PTSD compared three concurrent task manipulations: memory recall plus eye movements, memory recall plus alternate auditory tones, and memory recall in isolation. Findings indicated that memory recall plus eye movements reduced emotionality and vividness to a greater extent than memory recall plus tones and memory recall only. The authors argued that given the evidence, the use of tones as an alternative to eye movements within Eye Movement Desensitization and Reprocessing (EMDR) therapy was premature (Van den Hout et al., 2012). These studies exemplify Van den Hout's mastery and commitment in using experimental findings to inform clinical practice.

According to a WM account, simultaneously recalling an emotional memory and making eye movements compete for limited WM resources leaving less capacity for the memory resulting in it becoming less vivid and emotional (Gunter and Bodner, 2008, Van den Hout and Engelhard, 2012, Van den Hout et al., 2011). Van den Hout and Engelhard (2012) have reviewed experiments investigating the impact of various cognitive tasks on the recall of negative memories. As a variety of tasks, not just eye movements, was found to exert similar effects, the authors proposed that any task appropriately taxing WM should attenuate the vividness and emotionality of the memory (see also Gunter & Bodner, 2008). It is noted that an alternative account (cf. Baddeley & Andrade, 2000) has been proposed in earlier studies (e.g. Andrade et al., 1997, Van den Hout et al., 2001) hypothesizing the locus of the effect to be modality specific, e.g. via competing resources between visual WM task with visual aspects of the memory within the visuospatial sketchpad. This modality-specific proposal has been favoured to explain related findings from our lab in which engagement in a visuospatial WM task leads to a reduction in visual intrusive memory frequency after an experimental trauma (a traumatic film) as compared to verbal WM tasks (e.g. Bourne et al., 2010, Holmes et al., 2010), at least in some studies.

Results from different experimental paradigms may explain the discrepant views. While Van den Hout and colleagues research generally models voluntary retrieval of autobiographical memories with vividness/emotionality as outcome measures, our work above has modelled involuntary intrusive memories to trauma films and their frequency. Further research is needed to determine the precise WM mechanisms of emotional memory interference across paradigms and emotional memory types. Nevertheless, such paradigms are united in an interest in understanding cognitive mechanisms that might underlie the modulation of memory following trauma exposure. Furthermore, both rely on the notion of interference effects, and for both the temporal constrains of memory interference remain to be tested.

We have sought to test whether visuospatial WM tasks can reduce intrusive memories using the trauma film paradigm (e.g. Holmes et al., 2009, Holmes et al., 2010). In this laboratory-based paradigm, participants view short films containing scenes depicting traumatic events which involve actual/threatened death or serious injury, as an experimental analogue of viewing a traumatic event (American Psychiatric Association, 2013). Subsequently, the occurrence of intrusive memories is recorded, over the course of 1-week in daily life, or on a laboratory intrusion provocation task. Visuospatial WM tasks (such as tapping a complex sequence on a keypad held out of sight) performed *during* viewing of traumatic film footage, i.e. *concurrent* memory interference, reduce the number of image-based intrusive memories of the film relative to non-visuospatial control conditions (Bourne et al., 2010, Holmes et al., 2004, Stuart et al., 2006). Further, a visuospatial WM task performed immediately after film viewing, i.e. *retroactive* interference, show the same pattern of results (Deeprose et al., 2012). A cognitive task procedure involving a brief reminder task for the film followed by playing the visuospatial-based computer game ‘*Tetris*’ (Pajitnov & Nelson, 2008) relative to a non-visuospatial-based computer game, some time after exposure to traumatic film footage also reduces intrusive memory frequency over the subsequent week (e.g. when played 30 min post-film, Holmes et al., 2009; and 4 h post-film, Holmes et al., 2010).

We have interpreted such findings as follows: after an event, memory undergoes a time-dependent process of stabilization, termed consolidation (McGaugh, 2000). During this period of consolidation, memory is fragile and vulnerable to interference (Nader et al., 2000, Walker et al., 2003). Visuospatial WM tasks presented concurrently or after experimental trauma, while memory is undergoing consolidation, are hypothesised to interfere *concurrently* or *retroactively* (Dewar et al., 2007, Wixted, 2004, Wixted, 2005) with visual representations of the trauma film, resulting in a reduction in subsequent intrusive memories to the film (e.g. Holmes et al., 2009).

Thus far, our research has focussed exclusively on the use of WM tasks presented either concurrent with or soon after experimental trauma (Deeprose et al., 2012, Holmes et al., 2009, Holmes et al., 2010). However, research suggests that memory interference can also occur *proactively*, as defined above (Wixted, 2004, Wixted, 2005). Proactive interference has been proposed to be the main cause of normal forgetting in everyday life (Underwood, 1957). Experimentally, proactive interference in memory has been observed using a range of paradigms, such as masking (Feredoes et al., 2006, Hartshorne, 2008), ‘AB–AC’ paired-associate cued recall (e.g. Henson, Shallice, Josephs, & Dolan, 2002), and Pavlovian learning paradigms (Bouton, 1993). Proactive interference effects have also been observed for high and low similarity items over the course of several days using word list discrimination tasks (for example; 7 days, Postman, 1962; 1 day, Underwood, 1957) and different tests of memory (recognition memory and recall memory tests completed consecutively over 2 days; Lustig & Hasher, 2002). The use of discrimination tasks have also been combined with dual task paradigms (where the simultaneous task is finger sequence tapping) demonstrating that dual tasks conditions led to an increase in proactive interference (Kane & Engle, 2000).

Proactive interference on emotional memory has also been investigated. For example, following repeated learning of emotional and neutral pictures and their specific spatial location on a computer screen, more recall mistakes were made for locations of emotional compared to neutral items, indicating that emotional items were more susceptible to proactive interference (Novak & Mather, 2009). Further, memory for novel associations between cues and contextual details was poorer when such cues had been previously associated with emotional as opposed to neutral items, again consistent with proactive interference effects (Mather & Knight, 2008). Using the trauma film paradigm to specifically study *intrusive* emotional memories, a relationship was found between two separate measures, one indexing individual differences in the ability to resist proactive interference and another measuring intrusion frequency following a trauma film (Verwoerd et al., 2011, Wessel et al., 2008). However, rather than studying separate tasks, the actual impact of a proactive WM task interference *on* intrusive emotional memory does not appear to have been examined.

We suggest that proactive interference effects on intrusive memory could be tested within the trauma film paradigm, using the type of cognitive task already known to interfere concurrently or retroactively with the film. Our previous studies have shown large effects on reducing intrusions when tasks were undertaken concurrently to (d = 0.80, Bourne et al., 2010) or 30 min after (d = 0.91; Holmes et al., 2009) trauma-film viewing. This suggests that the cognitive tasks exerted effects through concurrent or retroactive interference with the trauma-film memory. The cognitive tasks used were visuospatial – complex concealed pattern tapping or *Tetris* game play respectively. Memory interference theory suggests that interference from competing information (here the film and the task) may occur irrespective of the order of either event (Wixted, 2004). However, the impact of *proactiv*e interference (i.e. a task before trauma film viewing) remains to be tested.

The current study examined whether a visuospatial WM task (*Tetris* game play) played immediately *prior* to viewing experimental trauma (a film) would reduce subsequent intrusive memories of the film over one week, consistent with the idea that a WM task could proactively interfere with the consolidation of intrusive memories. Our power calculation for sample size assumed a large effect size for comparability to those studies in which visuospatial tasks were undertaken concurrently (Bourne et al., 2010) or soon after (Deeprose et al., 2012, Holmes et al., 2009, Holmes et al., 2010) experimental trauma. To recap, this question holds importance both theoretically and clinically. It allows us to examine whether theoretical predictions concerning proactive memory interference extend to intrusive emotional memory and using the trauma film paradigm (rather than just static emotional images). Given that there are many situations in which trauma are predictable, a preventative intervention which could be delivered *prior* to entering a traumatic situation would be useful.

We hypothesised that participants assigned to a cognitive task condition in which the computer game *Tetris* is played immediately *before* viewing traumatic film footage, relative to participants in a No-Task Control condition (participants *do not* play *Tetris* but instead sit quietly for the equivalent period of time prior to the film), would report:

i. Fewer intrusive memories of the film over the subsequent week in daily life, as recorded in an intrusion diary.
ii. Fewer intrusions in response to an Intrusion Provocation Task (IPT) administered at 1-week in the laboratory.

## 2 Materials and methods

### 2.1 Participants

Fifty six healthy volunteers (32 females) took part in the study and were remunerated for their participation. Participants were recruited from two university campuses and from the general public via advertisements in an online newspaper and in the community. Participants were eligible to take part if they; *a*) were between the ages of 18 and 65 years of age, *b*) reported no mental health problems, and *c*) were not familiar with the study or had not taken part in a study of a similar nature. Ethical approval was obtained from the University of Oxford Central University Research Ethics Committee [MSD/IDREC/C1/2011/102].

### 2.2 Study stimuli

#### 2.2.1 Trauma film

The 12 min trauma film contained 11 scenes depicting actual or threatened death, and serious injury as an experimental analogue of viewing a traumatic event (DSM-5; American Psychiatric Association, 2013). Each scene contained footage with different content e.g. a man drowning in the sea; a young girl hit by a car with blood dripping out of her ear. Film footage was presented on a 17 inch colour monitor. The film had been used in previous studies to induce image-based intrusions (Holmes et al., 2009; Exp 2 from Holmes et al., 2010, James et al., 2015).

### 2.3 Task

#### 2.3.1 Tetris – a visuospatial WM task

The visuospatial-based computer game *Tetris* (Pajitnov & Nelson, 2008) involves manipulating a series of seven differently coloured and shaped 2-D geometric blocks using the arrow cursor keys on a standard keyboard. Blocks fall at a steady pace one at a time from the top of the playing screen in a random order. As they fall they can be moved from left to right and rotated 90°. The aim of the game is to place the blocks as they fall in such a way as to form continuous horizontal rows at the bottom of the screen. Participants were encouraged to use mental rotation when the blocks were falling. When a continuous row is made it is removed from the screen and the player is awarded points. Over the course of the game as more rows are completed the blocks descend faster. Participants played *Tetris* on a PC with no sound using the same monitor as film viewing.

### 2.4 Self-report questionnaires

#### 2.4.1 Baseline measures

##### 2.4.1.1 Beck Depression Inventory-II (BDI-II)

Depressed mood was measured using the BDI-II (Beck, Brown, & Steer, 1996). The BDI-II has 21 self-report items each measured on a scale of 0–3. Scores range from 0 to 63, where higher scores indicate greater levels of depression. The BDI-II shows high internal validity, α = 0.81 (Beck, Steer, & Garbin, 1988).

##### 2.4.1.2 State Trait Anxiety Inventory – Trait (STAI-T)

Trait anxiety was measured using the STAI-T (Spielberger, Gorsuch, Lushene, Vagg, & Jacobs, 1983). The item has 20 items and contains several anxiety-absent items (e.g. *I am content*) that are reverse scored. Scores range from a minimum of 20 to a maximum of 80 with higher scores representing greater levels of trait anxiety. The STAI-T demonstrates good internal validity; α = 0.90 (Speilberger, Reheiser, Owen, & Sydeman, 2004).

##### 2.4.1.3 Spontaneous Use of Imagery Scale (SUIS)

An individual's tendency to engage in spontaneous mental imagery in their day-to-day lives was measured using the SUIS (Reisberg, Pearson, & Kosslyn, 2003). Participants rate on a scale of 1 (*never*) to 5 (*always*) 12 items including; ‘*When I hear a radio announcer or DJ I've never actually seen, I usually find myself picturing what they might look like*’. Scores range from a minimum of 12 indicating ‘*no use*’ to 60 ‘*high use*’. The SUIS has been shown to have excellent internal consistency (α = 0.98; Reisberg et al., 2003).

##### 2.4.1.4 Eysenck Personality Questionnaire – Neuroticism scale (EPQ-N)

The EPQ-N (Eysenck, Eysenck, & Barrett, 1985) contains 12 items. Each item requires a ‘*yes*’ or ‘*no*’ response. Positive scores are summed and range from zero indicating low neuroticism to 12 indicating high neuroticism. The EPQ-N has good internal validity; α = 0.84 (Eysenck et al., 1985).

##### 2.4.1.5 Traumatic Experience Questionnaire (TEQ)

(Adapted from Foa, Ehlers, Clark, Tolin, & Orsillo, 1999). Participants reported their prior trauma history using a 12-item checklist adapted from criterion A of the Posttraumatic Diagnostic Scale (Foa, 1995). Participants indicated whether they had experienced or witnessed each of a series of traumatic events. ‘*Yes*’ scores were summed and could range from 0 (no traumatic event) to 12 (each and every type of traumatic event experienced or witnessed).

#### 2.4.2 Film-related mood and distress ratings

##### 2.4.2.1 Pre- and post-film mood

Participants rated their levels of sadness, hopelessness depression, fear, horror, and anxiousness on six visual analogue scales (VAS) given both pre- and post-film. The VAS instructed participants to rate how they felt ‘*right at this very moment*’ and scales were anchored at one end with ‘*not at all*’ and the other ‘*extremely*’. A composite mood score was calculated by summing the six scales (James et al., 2015).

##### 2.4.2.2 Film distress

Participants rated their distress in relation to the film after viewing had ended. An 11-point scale was used anchored from 0 (*not at all*) to 10 (*extremely*).

### 2.5 Manipulation checks

#### 2.5.1 Film attention

Participants were asked to rate ‘*how much attention did you pay to the film you just watched*’ on an 11-point scale ranging from 0 (*not at all*) to 10 (*extremely*).

#### 2.5.2 Demand rating

All participants responded to the question, ‘*How much do you predict that playing the game Tetris before viewing a distressing film (rather than watching it normally) would increase or decrease intrusive images of the film of the type you recorded in your diary?*’ using a single VAS ranging from −10 (*extreme decrease*) to 10 (e*xtreme increase*) (based upon Holmes et al., 2004, Stuart et al., 2006) administered at the end of the second session on Day 7.

### 2.6 Intrusive memory measures for the trauma film

#### 2.6.1 Intrusion diary

Participants were asked to keep a pen and paper, tabular diary of any image-based intrusive memories of the trauma film they experienced in their daily lives in the 7 days after the film viewing, based upon that used in previous studies (Holmes et al., 2004, Holmes et al., 2009, Holmes et al., 2010, James et al., 2015). Intrusive memories were described as ‘mental images’ (e.g. “*in the form of pictures in your mind's eye*”) and were defined as being spontaneous memories of the film that occur without deliberate recall. Participants noted down in the diary every time they had a spontaneous image of the film come to mind and were asked to briefly note the content of each intrusive memories (in order for it to be later matched to the film in content). Written and verbal instructions were provided with the diary.

##### 2.6.1.1 Diary accuracy

A single VAS requested participants to ‘indicate how accurate you think your diary is’ on a scale from 1 (not at all accurate) to 10 (extremely accurate).

#### 2.6.2 Intrusion Provocation Task (IPT)

In the laboratory-based IPT (adapted from James et al., 2015, Lang et al., 2009, Malik et al., 2014), participants viewed 11 blurred (using GIMP (2010) software, Gaussian Blur 2.0) static visual images – one from each scene of the trauma film. The images were presented for 2 s each on a 17 inch colour monitor using PowerPoint slideshow. Immediately afterwards participants had a 2 min break during which they were asked to sit with their eyes closed and press a specific key on a computer keyboard whenever they had an intrusive memory of the film.

### 2.7 Recognition memory

A verbal recognition memory test comprised 32 written statements regarding the film (as used in Holmes et al., 2009; Exp 2 Holmes et al., 2010). There were approximately three statements per film clip Participants were asked to evaluate each statement as true or false based on their memory of the film. Example statements include; ‘*the bones in the girl's legs are seen to fall back into place*’ (True/False) or ‘*four small circles are made on the surface of the eye*’ (True/False).

### 2.8 Data analysis

All data were examined for potential univariate outliers. No scores were more than 3 standard deviations away from the mean, and therefore no data were classified as outliers (Tabachnick & Fidell, 1996). Independent samples *t*-tests were used to compare means between conditions for baseline measures (BDI-II, STAI-T, SUIS, EPQ-N and TEQ), film distress ratings, manipulation checks (film attention and demand ratings), intrusive memory measures for the trauma film (intrusion diary frequency of intrusions, diary accuracy and IPT intrusion frequency) and recognition memory scores; repeated measures ANOVAs were used to compare mood from pre-to post-film. Nominal data were analysed using the chi-square test. All statistical tests were two-tailed and used an alpha level of 0.05.

#### 2.8.1 Sample size estimation

Based on effect sizes of *d* = 0.91 found by Holmes et al. (2009; in which participants performed the *Tetris* task 30 min post-film viewing) and *d* = 0.80 found by Bourne et al. (2010; in which participants completed a visuospatial tapping task during trauma film viewing), the current study assumed a large but more conservative effect size of 0.8. A minimum sample size of *N* = 26 per condition is required to ensure an 80% power to detect this difference at the 5% significance level.

### 2.9 Procedure

**Fig. 1:**
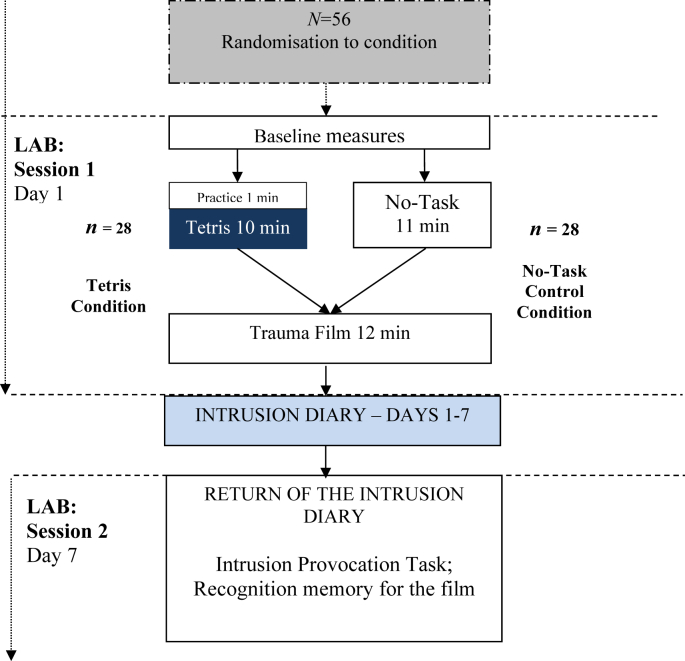
Procedural overview.

Participants completed 2 sessions, 7 days apart, and were tested individually. The experimenter was present for all procedures except film viewing. See Fig. 1 for a procedural diagram.

*Session 1* (*Day 1*). Upon arrival at the laboratory participants gave written informed consent and then completed pen and paper baseline measures. Following this, all participants in the *Tetris* condition completed a 1 min practice session of the computer game *Tetris* with experimenter guidance, followed immediately by a further 10 min play with no guidance. Participants in the *Tetris* condition were instructed to play using their dominant hand. Participants in the No-Task control condition sat quietly for an equivalent time period (11 min). Participants then watched the trauma film in a darkened room with the experimenter absent. Visual Analogue Scale (VAS) mood evaluations were taken before and immediately after film viewing. Attention paid to the film and distress ratings were also given after film viewing. Participants were then provided with detailed instructions (both verbally and written) on how to keep the intrusion diary over the course of the subsequent 7 days and were asked to return to the laboratory in 7 days.

*Session 2* (*Day 7*). One week later participants returned to the laboratory with their intrusion diaries. Participants completed a verbal recognition memory test for the film and underwent the Intrusion Provocation Task. Finally participants completed demand and diary accuracy ratings, and were debriefed at the end of the experiment.

## 3 Results

### 3.1 Baselines measures

There were no significant differences between conditions for baseline measures (Table 1).

### 3.2 Film related mood and distress ratings

#### 3.2.1 Mood pre-to post-film

Repeated measures ANOVA confirmed a main effect of time, indicating participants' mood significantly deteriorated following the film. There was no main effect of group, or a group × time interaction. There were no significant differences between conditions for ratings of film distress (Table 2).

### 3.3 Intrusive memory and recognition memory in relation to the trauma film

There was no statistically significant difference in the number of intrusive memories reported in the diary in the week following trauma film viewing, or the IPT given at 1-week between conditions. Further, participants were comparable for self-rated measures of diary accuracy. Scores on the verbal recognition memory test for the film did not differ significantly between conditions (Table 3).

### 3.4 Manipulation checks

#### 3.4.1 Attention to film and demand rating

Participants did not significantly differ between conditions on ratings of attention paid to the film or experimental demand (Table 4).

## 4 Conclusion and discussion

The current experiment reports null findings, with potential clinical and theoretical relevance. Results failed to support our initial hypothesis: i.e. compared to a control condition, participants who played the computer game *Tetris* immediately *before* viewing traumatic film material did not report statistically significantly fewer intrusive memories of the film in the subsequent week as measured by (i) the intrusion diary in daily life or (ii) the Intrusion Provocation Task (IPT) at the 1-week follow up in the laboratory. The estimates of effect size for each intrusion measure were small, as shown by the size of the Cohen's *d* statistic. Thus, current results using a proactive interference design stand in contrast to the large effect sizes found in concurrent/retroactive interference studies with a trauma film paradigm (Bourne et al., 2010, Holmes et al., 2009). While it is possible there is a small effect of *Tetris* on reducing diary intrusions when played before the trauma film, corresponding to our *d* value of 0.26, future studies would be required to test this a priori. To have sufficient power to detect such an effect a sample of *N* = 468 would be required. Nevertheless, if this was the case such a reduction might suggest clinical potential given that such an intervention would be relatively low intensity to deliver. Overall, conclusions should be drawn with caution and current results suggest that a from an experimental psychopathology perspective, interventions to reduce intrusive memories using a WM interference rationale may not be as effective if administered pre-trauma, compared to the same cognitive task administered during (Bourne et al., 2010) or after trauma (Deeprose et al., 2012, Holmes et al., 2009).

Participants in *Tetris* and No-Task Control conditions were matched at baseline for levels of depression, trait anxiety, general imagery use, neuroticism and prior traumatic history. Moreover, change in mood pre- and post-film, attention paid to the film, distress relating to the film, diary accuracy, and recognition memory scores of the film at 1-week follow-up were not significantly different between conditions, suggesting that these factors are also unlikely to account for the current null findings.

Results also raise questions about the extension of proactive interference predictions from the non-clinical memory literature to emotional intrusive memory. The memory interference literature assumes interference will occur regardless of the order of the presentation tasks (Wixted, 2004). Thus we had hypothesised that *Tetris* played *before* traumatic film viewing may interfere with the processing of traumatic film content. However, current results failed to support this in relation to a statistically significant difference in the frequency of intrusions between groups. Why might this be? One argument from the memory interference literature would be that current findings may be due to the lack of *similarity* between the computer game *Tetris* and the film footage thus yielding insufficient interference. Interference stimuli used in the masking literature (e.g. Hartshorne, 2008) share similar properties such as category relatedness (e.g. same material type) and spatial similarity (e.g. shared location). Early studies showed proactive interference across variety of paradigms, even with relatively low similarity and with temporal variation (Underwood, 1957). More recent evidence suggests similarity-based interference is more effective (e.g. Bunting, 2006), though some instances of proactive interference have also been observed in paradigms with low similarity. For example, Lustig and Hasher (2002) used non-identical memory tasks performed on consecutive days to show proactive interference effects on a subsequent working memory span task 24 h later. However, we note this broader issue remains a puzzle to explore further since the Tetris task appeared sufficient in similarity to be able to retroactively interfere with the film (e.g. Holmes et al., 2009, Holmes et al., 2010), yet not so proactively.

Further, there are features which, we propose, make *Tetris* a good candidate for interfering with intrusive memories of the film. Shared features include the visuospatial nature of the different coloured, moving 2D blocks that comprise the game *Tetris* (Green & Bavelier, 2003) in relation to the colour 2D moving film over time, delivery on the same computer screen and so forth. Furthermore, the same task has been shown sufficient to interfere with the same film when delivered at other time frames. Yet, while this possibility seems unlikely there are other features such as the dynamic nature of Tetris game play and the film footage, which differ from kind of static images used more typically in proactive interference research (e.g. Novak & Mather, 2009), which could also contribute to current findings.

The interference literature also suggests temporal contingencies may be a factor influencing proactive interference. Thus one account for our findings may be that the Tetris game and film viewing were relatively as far apart in time, e.g. several minutes, compared to typical proactive interference research methods (in which the inter stimuli timing is milliseconds).

Another factor might be the duration of task relative to the film duration. Potentially, increasing the duration of pre-film *Tetris* game play could enhance its ability to interfere with consolidation of the 12 min trauma film content by increasing the extent of visual memory resources dedicated to representations of game play. The current play duration of 11 min was equivalent to studies in which *Tetris* was played *after* viewing a traumatic film but could be varied. Interestingly the ‘*Tetris* effect’ (Stickgold, Malia, Maguire, Roddenbury, & O'Connor, 2000) is a term given to the phenomenon whereby visual representations of *Tetris* persist in mind following extended periods of game play (for example, 1 h of game play, twice a day). Further, it has been shown that *Tetris*-themed memory traces interacted with remote autobiographical memories that were not of *Tetris* (Stickgold et al., 2000). Increasing the duration of pre-trauma *Tetris* game play to a level at which participants report experiencing the ‘*Tetris* effect’ (e.g. 1–2 h) might increase the impact on intrusive memories, although one might advise caution against excessive computer game play.

Other possible explanations for current findings relate to the degree of competition between prior *Tetris* game play (when in the form of a memory representation rather than actual game play) and the *emotional* nature of the film. Incoming emotional perceptual content “hijacks” attention (LaBar and Cabeza, 2006, Vuilleumier, 2005). Thus, the emotional content of the traumatic film may have overridden the ability of *Tetris*, when played *prior* to trauma film viewing, to be retained in short term memory to a sufficient degree to interfere with trauma film content. Another difference with our previous studies in which playing *Tetris after* a trauma film reduced later intrusive memories (e.g. Holmes et al., 2010), is that visual representations of emotional traumatic film content purportedly underwent interference from *actual* game play, rather than a *memory representation* of the game. Perhaps this alongside the emotionality of the film could weaken interference effects.

The current study focussed on proactive interference effects on intrusive memory. In addition, there was no statistically significant difference between conditions on recognition memory (*d* = 0.04). Interestingly, a previous study employing concurrent interference has shown a discrepancy between intrusive versus voluntary memory (Experiment 2, Bourne et al., 2010). We note this pattern challenges mainstream memory theories (e.g. Tulving, 2002) which would predict that disrupting memory consolidation would impact both involuntary and voluntary memory which are from the same underlying memory trace. However, our current study showed that a WM task administered to interfere proactively with the film did not impact either involuntary intrusions or a recognition test, suggesting neither forms of memory are disrupted.

Finally, we note that our previous studies used trauma-film reminder cues (memory reactivation task) prior to *Tetris* game play (Holmes et al., 2009, Holmes et al., 2010, James et al., 2015). Clearly one cannot deliver a reminder of a film before the film has been viewed. However, it is possible that reminders of the *Tetris* task during film viewing may have aided proactive interference effects. Recent work has shown that a that a cognitive procedure involving *both* a memory reactivation ask and *Tetris* game play is critical for reducing intrusions within a memory *re*consolidation time-frame (e.g. when memory for the trauma film has consolidated >24 h old, James et al., 2015). The role of reminder cues (either for task or film), when using WM task interference procedures, should be further investigated.

Overall, on the basis of current evidence, these results permit the conclusion that a WM task of sufficient efficacy when presented after an experimental trauma (as in prior experiments) is not similarly efficacious when presented before an experimental trauma (the current experiment) to reduce intrusions. Hence, a critical question for further research is to delineate the optimal time frames for applying competing cognitive task procedures within a WM interference framework.

### 4.1 Limitations

The current study used an experimental trauma manipulation (viewing traumatic film footage) as an analogue for psychological trauma and subsequent intrusive memory symptoms in a healthy population. A common critique levied at the trauma film paradigm concerns whether indirect trauma in the form of film footage is sufficient to induce a traumatic response akin to that of ‘real-life’ trauma exposure. Recent guidelines in the Diagnostic and Statistical Manual of Mental Disorders state that exposure to trauma through electronic media, television and movies in the line of work (e.g. police officers) can be considered a ‘traumatic event’ (American Psychiatric Association, 2013). In addition, research investigating the impact of electronic media exposure on acute stress symptoms found that individuals who viewed extended media footage of the Boston Bombings (approximately 6 h), showed increased acute stress symptoms, compared to individuals who experienced actual, ‘real life’ exposure to the same event (Holman, Garfin, & Silver, 2014). Taken together film footage, as in the kind used in the current study, may be a useful analogue method to prospectively study traumatic reactions and subsequent symptoms in experimental settings. The trauma film paradigm is now widely used by other researchers to investigate how individuals process (analogue) trauma and subsequent PTSD-like symptoms, including Van den Hout, to whom this special issue is addressed (Kindt et al., 2008).

The current study did not contain a self-report rating of task compliance following *Tetris* game play and the no-task control condition, which should be added to future studies. The addition of manipulation measure to check whether memory representation of Tetris persisted beyond game play or not would allow us to tease apart whether Tetris did not provide interference, or whether interference just did not impact intrusion frequency, should be included in future studies. Further, it would be worthwhile to include baseline measures of WM capacity in future studies to investigate the impact of individual differences in WM on interference susceptibility and subsequent intrusion development.

### 4.2 Conclusion

Previous studies have shown that engaging in tasks that have a WM component, for example those involving eye movements, either during or after autobiographical memory recall (e.g. Engelhard et al., 2010, Van den Hout and Engelhard, 2012) reduces the vividness/emotionality of those emotional memories. Relatedly, a procedure involving playing the computer game *Tetris* after traumatic film viewing (e.g. Holmes et al., 2009) reduces the frequency of later intrusive memories of that film. While such studies tested possible concurrent or retroactive memory interference effects, the possibility of *proactive* memory interference (Wixted, 2004, Wixted, 2005) remained to be tested. The current study therefore changed the temporal order of events such that the WM task (here *Tetris* game play) occurred prior to the analogue trauma exposure (film viewing). The current lack of statistically significant differences in intrusive memories between conditions is of clinical and theoretical interest. Results suggest the same cognitive task which was successful in reducing intrusions when delivered *after* analogue trauma exposure is not as effective as when administered *prior* to exposure. This has relevance for the development of preventative interventions for those clinical groups in which trauma can be predicted to occur. Results flag temporal constraints on the delivery of memory interference tasks for intrusive memories of emotional events, suggesting further work is needed to bridge the non-clinical and clinical literature. These divergent results between studies and methods raise intriguing questions about boundary conditions of memory interference mechanisms in relation to intrusive emotional memory.

Ultimately, the effect of *Tetris* game play on intrusion frequency remains to be empirically tested within a clinical setting in real life, for example with frontline responders who are repeatedly exposed to traumatic events in the line of work. We note that a current study is underway (ClinicalTrials.gov Identifier: NCT02080351; 2015) testing the retrospective impact of *Tetris* am emergency department with people who have experienced a road traffic accident. The results of future clinical investigations should seek to assess not only the potential benefits of an intervention but also possible negative side effects (Jonsson, Alaie, Parling, & Arnbert, 2014). As a hypothetical example (given current results), if for emergency personnel, it were possible that proactive interference reduced intrusive memories, then perhaps proactive interference could also interfere with other processes that one would not want to interfere with such as the need by emergency workers to use visuospatial capacity to quickly make decisions in their work setting, and this should be investigated.

The current experiment is just one example of how our research in experimental psychopathology has been influenced by the elegant studies on trauma and WM led by Marcel Van den Hout. Combining his clinical and experimental expertise, Marcel has made an unrivalled contribution to our understanding of the role of eye movements in the effective, but little understood treatment for PTSD – EMDR. His legacy creates numerous broader sources of inspiration too, including the investigation of additional WM tasks to help ameliorate unwanted negative, emotional memories and the further development of experimental paradigms through which to continue to explore the mechanisms underlying changes in related psychopathologies after trauma.

## Figures and Tables

**Table 1:** Age, gender, baseline mood and anxiety measures, general imagery use, neuroticism and trauma history ratings for each experimental condition.

Measure	Tetris (*N* = 28)		No-task control (*N* = 28)		Analysis		
	***n***	**%**	***n***	**%**	**χ**^**2**^	**df**	***p***
Female	13	46.4	19	67.9	2.63	1	0.11
	***M***	***SD***	***M***	***SD***	***t*(54)**		***p***
Age (years)	20.46	2.03	20.71	1.54	0.52		0.61
BDI-II	7.14	7.07	7.07	5.63	0.04		0.97
STAI-T	39.64	11.39	36.32	9.66	1.18		0.25
SUIS	35.86	7.28	38.79	7.69	1.46		0.15
EPQ-N	4.86	3.27	4.64	2.16	0.29		0.77
TEQ	0.89	0.83	1.07	1.15	0.67		0.51

*Note*. BDI-II = Beck Depression Inventory – II (Beck et al., 1996); EPQ-N = Eysenck Personality Questionnaire – Neuroticism Scale (Eysenck et al., 1985); STAI = State Trait Anxiety Inventory – Trait (Spielberger et al., 1983); SUIS = Spontaneous Use of Imagery Scale (Reisberg et al., 2003); TEQ = Traumatic Experiences Questionnaire (adapted from Foa et al., 1999).

**Table 2:** Mood change pre- to post-film and film distress for each experimental condition.

Measure	Tetris (*N* = 28)		No-task control (*N* = 28)		ANOVA			
	**M**	**SD**	**M**	**SD**	**Time**	**Group**		Group*Time
Pre film mood VAS	8.93	10.00	7.55	4.52	F_(1,54)_ = 68.16^**§**^	F_(1,54)_ = .08		*F*_(1,54)_ = 0.24
Post film mood VAS	21.11	11.66	21.25	12.82
					***t***_**(54)**_		***p***	
Film distress	7.04	1.95	7.25	2.01	0.40		0.69	

*Note*. VAS (Visual Analogue Scale) mood composite sum of Sadness, Hopelessness, Depressed, Fear, Horror and Anxiousnes. ^**§**^*P* ≤ .001.

**Table 3:** Intrusive Memory, Diary accuracy and Recognition Memory for the Trauma Film.

Measure	Tetris (*N* = 28)		No-task control (*N* = 28)		Analysis		
*M*	*SD*	*M*	*SD*	*t*_(54)_	*p*	*d*
Intrusive memory frequency in diary	4.86	3.70	6.00	4.91	0.98	0.33	0.26
Intrusive memory frequency during IPT	5.00	3.68	4.57	2.78	0.49	0.63	0.13
Verbal recognition memory test score	19.89	3.61	19.75	3.00	0.16	0.87	0.04
Diary accuracy	7.57	1.87	7.82	1.76	0.51	0.61	0.14

Note. IPT = Intrusion Provocation Task.

**Table 4:** Manipulation checks for each experimental condition.

Measure	Tetris (*N* = 28)		No-task control (*N* = 28)		Analysis	
*M*	*SD*	*M*	*SD*	*t*_(54)_	*p*
Attention to film	9.11	1.03	9.32	0.77	0.88	0.38
Demand rating	0.29	3.56	0.11	4.24	0.17	0.87
